# Effects of 5′-3′ Exonuclease Xrn1 on Cell Size, Proliferation and Division, and mRNA Levels of Periodic Genes in *Cryptococcus neoformans*

**DOI:** 10.3390/genes11040430

**Published:** 2020-04-16

**Authors:** Xueru Zhao, Xin Li, Ping Zhang, Chenxi Li, Weijia Feng, Xudong Zhu, Dongsheng Wei

**Affiliations:** 1 National Key Program of Microbiology and Department of Microbiology, College of Life Sciences, Nankai University, Tianjin 300071, China; zhaoxrw@163.com (X.Z.); lixinlixin1128@163.com (X.L.); 2120171026@mail.nankai.edu.cn (W.F.); 2 Beijing Key Laboratory of Genetic Engineering Drug and Biotechnology, Institute of Biochemistry and Molecular Biology, School of Life Sciences, Beijing Normal University, Beijing 100875, China; zp1516@163.com (P.Z.); lcx1219@163.com (C.L.)

**Keywords:** *Cryptococcus neoformans*, Xrn1, exonuclease, cell size, cell cycle, periodic genes

## Abstract

Cell size affects almost all biosynthetic processes by controlling the size of organelles and disrupting the nutrient uptake process. Yeast cells must reach a critical size to be able to enter a new cell cycle stage. Abnormal changes in cell size are often observed under pathological conditions such as cancer disease. Thus, cell size must be strictly controlled during cell cycle progression. Here, we reported that the highly conserved 5′-3′ exonuclease Xrn1 could regulate the gene expression involved in the cell cycle pathway of *Cryptococcus neoformans*. Chromosomal deletion of *XRN1* caused an increase in cell size, defects in cell growth and altered DNA content at 37 °C. RNA-sequencing results showed that the difference was significantly enriched in genes involved in membrane components, DNA metabolism, integration and recombination, DNA polymerase activity, meiotic cell cycle, nuclear division, organelle fission, microtubule-based process and reproduction. In addition, the proportion of the differentially expressed periodic genes was up to 19.8% when *XRN1* was deleted, including cell cycle-related genes, chitin synthase genes and transcription factors, indicating the important role of Xrn1 in the control of cell cycle. This work provides insights into the roles of RNA decay factor Xrn1 in maintaining appropriate cell size, DNA content and cell cycle progression.

## 1. Introduction

Cell size varies widely between different cell types, but it is narrowly distributed within specific cell types and under specific growth conditions [1]. Altering cell size has a significant impact on cell physiology, as it determines the size of organelles and influences surface transport [2,3]. Cell size control has been studied extensively in a number of different model organisms. In budding yeast *Saccharomyces cerevisiae*, size control occurs in the G1 phase before START, the point of irreversible commitment to cell division [4,5]. Growing budding yeast and primary mammalian cells with abnormally increased cell size are usually associated with impaired gene induction, abnormal cell cycle progression, and disordered cell signaling [1]. Maintenance of a cell type-specific DNA to cytoplasm ratio is essential for many cellular processes, and beyond this cell type-specific ratio contributes to senescence [1].

Messenger RNA turnover is a critical modulator of gene expression [6]. In general, the decay of most eukaryotic mRNAs occurs through three major pathways: deadenylation-dependent, deadenylation-independent and endonucleolytic cleavage-dependent [7]. Xrn1 is a highly conserved (from yeast to human) 5′-3′ exoribonuclease in the deadenylation-independent pathway and participates in diverse aspects of RNA metabolism such as nonsense-mediated decay (NMD), gene silencing, rRNA maturation, and transcription termination [7,8]. *S. cerevisiae* Xrn1 associates with Dcp1/Dcp2, a decapping enzyme that hydrolyzes the 5’ cap and exposes the mRNA to be cleaved by Xrn1 [9]. Mutation of *XRN1* in *S. cerevisiae* causes slower growth rates, increased cell size and changes in mRNA and protein levels [10], but the viability of the mutant cells is not affected [11]. In *Schizosaccharomyces pombe*, the deletion of *XRN1* leads to an increase in cell size at 20 °C and the mutant cell is sensitive to the cold stress [12]. Other phenotypes related to the deletion of *XRN1* include reduced rates of diploid formation [13], decreased sporulation [14], microtubule destabilization [11], nuclear fusion defect and chromosome loss [15], and deficiencies in filamentous growth [16,17]. The basidiomycetous yeast *C. neoformans* is a major human pathogen responsible for more than estimated millions of infections [18]. Three major virulence-associated factors have been established, including the ability to grow at 37 °C, the formation of polysaccharide capsule and the production of pigment melanin [19,20,21]. Deletion of cytoplasmic exonuclease *XRN1* in *C. neoformans* has been reported to lead to growth defects at 37 °C, smaller capsule, reduced melanin formation and impaired uni- and bisexual mating [22]. Here, we further find that the deletion of *XRN1* led to an increase in cell size, which was lethal when the temperature was set at 37 °C. The KEGG analysis of the transcriptome showed a significant difference in the cell cycle pathway between WT and mutant cells, and the expression of many genes involved in cell cycle progression was up-regulated in mutant cells. In addition, it was found that the deletion of *XRN1* disturbed the expression of many periodic genes. Our results showed a coordinated control of gene expression by *XRN1* to maintain proper expression of cell cycle related genes. This coordination may be important to control the cell size.

## 2. Materials and Methods

### 2.1. Strains and Culture Conditions

*Cryptococcus neoformans* var. *neoformans* serotype D uracil auxotrophic strain 4500FOA was the recipient strain used for deletion of *XRN1* in this study. Yeast strains were cultured at 28 °C on yeast extract peptone dextrose plates (YPD medium containing 2% glucose, 2% Bacto peptone and 1% yeast extract, pH 6.0). YNB plates used for the selection of *XRN1*-deletion transformants contained 0.17% yeast nitrogen base (YNB without ammonium and amino acids), 0.5% ammonium sulphate and 2% glucose (pH 6.0). The complementation strain was selected on YPD or YNB plates with 100 mg/L hygromycin. All vectors were amplified in *Escherichia coli* DH5α.

### 2.2. Construction of xrn1∆ and Complementation Strains xrn1∆-C

*XRN1* gene was deleted in the 4500FOA strain through CRISPR-Cas9 system. The suicide cassette for eliminating the CRISPR-Cas9 system was described previously [23]. Briefly, the pRH003 vector was used to construct the deletion vector (Figure S1a), which could produce gRNA and Cas9 nuclease in *C. neoforman*s. Target sequence (guiding RNA) was inserted into *Bsp*QI site by annealing two reverse complementary primers (TTG-N19/AAC-N19). Two homologous fragments were obtained by polymerase chain reaction and ligated to *Cla*I and *Bam*HI sites flanking the *URA5* gene using In-Fusion HD Cloning kits (Takara, Dalian, China). The Cas9 nuclease could create a double strand break in the red position and trigger double crossover by homology-directed repair (Figure S1a). The resultant plasmid pBS-ura5-Xrn1-Cas9-gDNA was digested with *BamH*I for linearization and transformed into 4500FOA strain by the electroporation method [23]. With *URA5* as the selection marker, transformants growing on YNB medium (without uracil) were selected for further genome verification.

To construct the complementation strain, a 7.4 kb fragment containing the wild-type copy of *XRN1* was obtained. This fragment was PCR amplified with high-fidelity polymerase from the wild-type genome using primer pair C-Xrn1-F/C-Xrn1-R. The *xrn1*∆ strain was co-electroporated with *XRN1* expression cassette and the linearized pBS-Hyg. Transformants were selected on YPD plates containing 100 mg/L hygromycin. The primers used in this study are listed in Supplementary Table S1.

### 2.3. Southern Blot Analysis

Overnight cultures (16 h) were harvested from the YPD liquid medium by centrifugation, and the genomic DNA was extracted and digested with *Eco*RV and *Sal*I. Probe was amplified from the wild-type genome using primer pair Xrn1-up-F/Xrn1-down-R and labelled with digoxigenin. A Southern blot procedure followed the protocol of DIG High Primer DNA Labeling and Detection Starter Kit II (Roche, Mannheim, Germany) as previously described [24].

### 2.4. Determining the Cell Size, Growth Curves and Budding Rate

For cell size measurement, yeast strains were cultured in the YPD liquid medium at 28 °C or 37 °C, with shaking speed of 180 rpm for 48 h. Cells were harvested and the cell size was measured with a Nikon Eclipse 80i fluorescence microscope (Nikon, Tokyo, Japan). Forty cells were removed randomly from each sample to measure cell size. Data analysis was performed using GraphPad Prism 5 software. Unpaired *t*-test was used for statistical analysis.

For growth curves, overnight cultures were transferred to fresh YPD liquid medium and continued to culture at 28 °C or 37 °C for 48 h. Absorbance was measured at 600 nm every 2 h, and the initial absorbance of OD_600_ was 0.2. Data were processed using GraphPad Prism 5 software.

For calculation of the budding rate, overnight cultures were transferred to the fresh YPD medium and continued to culture at 28 °C or 37 °C for 16 h (mid-log phase). The total number of yeast cells and the number of budding cells were counted under a microscope. The assay was performed three times. Budding rate was performed using GraphPad Prism 5 software. Unpaired *t*-test was used for statistical analysis.

### 2.5. Determining the Sensitivity of xrn1∆ Strain to Stress Conditions

Overnight cultures were harvested, diluted by gradient and spotted on YPD agar plates containing different kinds and concentrations of osmotic stabilizers, and the plates were incubated at 37 °C for 3 days. To test the sensitivity to nocodazole, yeast cells were spotted on YPD agar medium containing 0.4 μg/mL nocodazole and incubated at 28 °C for 3 days.

To further verify the sensitivity of *xrn1*∆ to nocodazole, cultures were grown in a YPD liquid medium with 1% dimethyl sulfoxide (DMSO) to early log phase (5 × 10^6^ cells/mL), and nocodazole was then added to a final concentration of 15 μg/mL and cells were further incubated at 28 °C for 5 h. Cells were harvested and observed under a microscope (Nikon, Tokyo, Japan).

### 2.6. Observation of Nucleus and Flow Cytometry

Overnight cultures were transferred to the fresh YPD liquid medium and continued to culture at 37 °C for 60 h. About 10^7^ cells were harvested and washed 3 times with PBS, fixed overnight with 70% ethanol at 4 °C, washed 3 times and resuspended in 2 mL sodium citrate buffer (20 mM sodium citrate, 50 mM EDTA, 0.45 mM sorbitol, 0.1% Triton-X100 and pH 5.5), incubated at 30 °C for 1 h. Cells were then treated with 40 μL RNase A (10 μg/mL) and incubated at 37 °C for 2 h, and further incubated with propidium iodide (PI, 30 μL, 1 mg/mL) at 37 °C for 10 min. The fluorescence was observed with a fluorescence microscope. The cells were analyzed by flow cytometry in a FACS Calibur flow cytometer (Becton-Dickinson, USA). Data were processed with the FlowJo V10 software.

### 2.7. RNA Preparation and Quantitative Real-Time PCR

Total RNA isolation was extracted with a RNAprep pure Plant Kit (Tiangen, Beijing, China). Semi-quantitative RT-PCR and qRT-PCR were performed as described previously [25]. Double-stranded cDNA was synthesized with cDNA Synthesis Kit (Takara, Dalian, China). qRT-PCR was performed on a StepOnePlus™ Real-Time PCR System with SYBR green master mix (Roche, Mannheim, Germany). ∆∆CT method was employed to assess transcriptional level of the target genes, and actin-encoding gene (*ACT1*) was used as the reference gene.

### 2.8. RNA-Sequencing Analyses

Yeast cultures were grown in YPD at 28 °C overnight, transferred to fresh YPD and incubated at 37 °C for 3 h. Yeast cells were harvested to isolate RNA using the TRIzol^®^ method. RNA sequencing was accomplished by a commercial service (The Beijing Genomics Institute, Beijing, China). The library was validated on Agilent Technologies 2100 bioanalyzer for quality control. The final library was amplified with phi29 (Thermo Fisher Scientific, Waltham, MA, USA) to make a DNA nanoball (DNB), which had more than 300 copies of one molecular; DNBs were loaded into the patterned nanoarray and single end 50 bases reads were generated on BGISEQ500 platform (BGI, Beijing, China). HISAT (Hierarchical Indexing for Spliced Alignment of Transcripts) software was used for alignment of the *C. neoformans* JEC21 genome and RNA-seq reads. The cor function in R Project was used to calculate the Pearson’s correlation between every two samples. Transcriptome results were submitted to the NCBI SRA database, and the BioProject accession number is PRJNA604887.

## 3. Results

### 3.1. Characterization of XRN1 Gene in C. Neoformans

**Figure 1:**
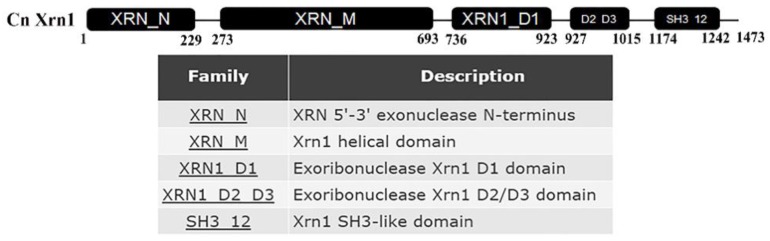
Conserved domain of Xrn1, which contained 5’-3’ exonuclease N-terminus, helical domain, D1 domain, D2/D3 domain and SH3-like domain.

Amino acid sequences of *XRN1* genes from *S. cerevisiae* were used to search the orthologues in *C. neoformans var. neoformans* serotype D JEC21. Only one homologous protein was found in *C. neoformans* (locus CNE03620, 39.84% identity) which contains 5’-3’ exonuclease N-terminus, helical domain, distinct domains (D1, D2/D3 domain) and Src homology 3-like (SH3-like) domain (Figure 1).

To investigate the function of Xrn1, we constructed Xrn1-deficient strain by replacing the genomic copy with Ura5 cassette via CRISPR-Cas9 system (Figure S1a). The CRISPR-Cas9 system includes gRNA-mediated Cas9 protein for cleavage of double-stranded DNA, and donor DNA from JEC21 genome for homologous recombination. The complementation strain was constructed by co-electroporating with *XRN1* cassette, and the linearized plasmid pBS-Hyg. 2029-bp and 2845-bp products (fragments A and B in Figure S1a) were obtained in the *xrn1*∆ mutant by PCR with two pairs of primers YZ-Xrn1-uup-F/YZ-Ura5-R and YZ-Ura5-F/YZ-Xrn1-ddown-R. However, no PCR products were amplified from the wild-type strain (Figure S1a). To further confirm the deletion of the *XRN1* gene, Southern blot was applied (Figure S1b). There were two signal bands on the membrane of the *xrn1*Δ strain, which are 2393 bp and 4373 bp, respectively. However, only one 6256-bp band was obtained in the WT, and three bands (2393 bp, 4373 bp, and 6256 bp) were observed in the complementation strains.

### 3.2. XRN1 Deletion Increased the Cell Size

**Figure 2:**
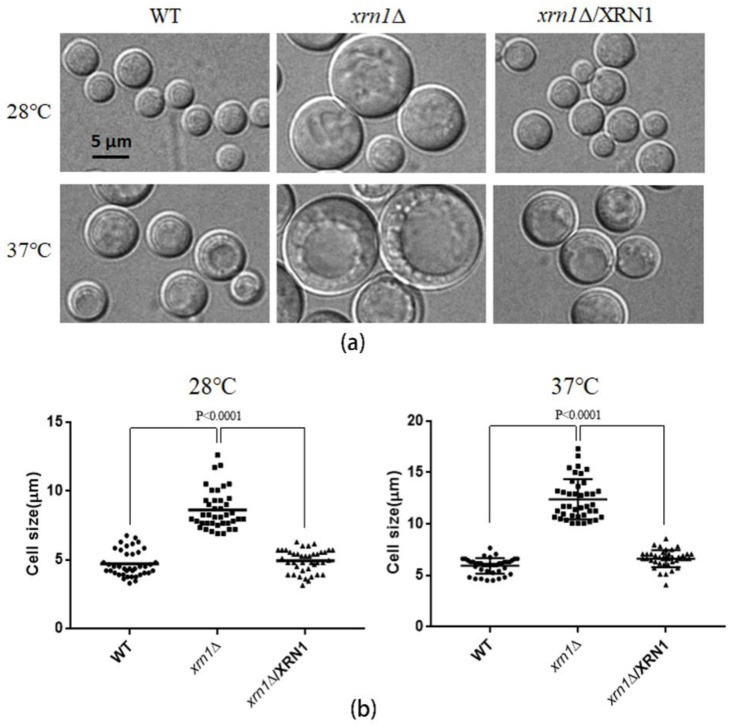
Measurement of the cell size. (**a**) Yeast strains were cultured in YPD liquid medium at 28 °C or 37 °C, with 180 rpm shaking for 48 h. Cells were harvested and observed with a Nikon Eclipse 80i fluorescence microscope (Nikon, Tokyo, Japan). (**b**). Forty cells were removed randomly from each sample to measure cell size. Data were processed using GraphPad Prism 5 software. Unpaired *t*-test was used for statistical analysis.

When the *xrn1*∆ mutant cells were cultured in YPD medium, it was found that the deletion of *XRN1* caused an increase in cell size under a microscope (Figure 2a). After 48 h of culture at 28 °C, the cell size of the *xrn1*∆ strain reached 8.62 ± 0.22 μm (Figure 2b). However, the cell size of the WT and complementation strains was 4.71 ± 0.15 μm and 4.92 ± 0.13 μm, respectively. This difference was more significant under 37 °C culture conditions, with a cell size of 12.40 ± 0.31 μm in the mutant and 5.91 ± 0.12 μm, 6.62 ± 0.13 μm in the WT and complementation strains, respectively. The size of the *xrn1*∆ mutant could reach 20 μm when we cultured yeast at 37 °C for a long time (Figure S2).

**Figure 3:**
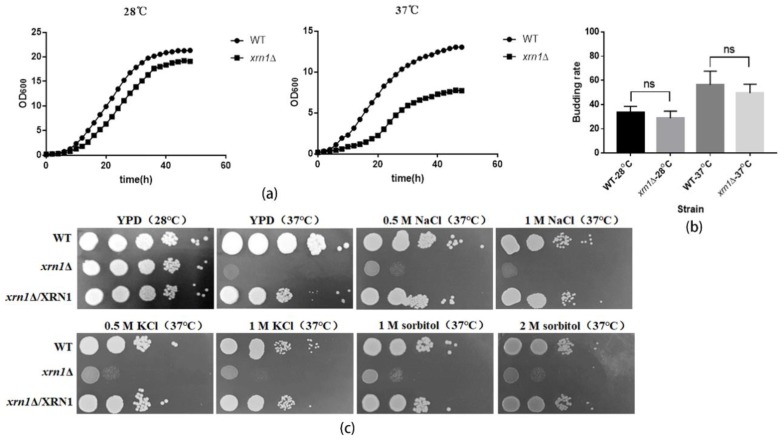
Growth defects of the *xrn1*∆ strain. (**a**) Determination the growth curves. (**b**) Budding rate of the WT and Xrn1-deficient strains. The number of budding cells per 100 cells was counted. Data analysis was performed using GraphPad Prism 5 software. Unpaired *t*-test was used for statistical analysis. (**c**) Growth of yeast cells at 28 °C or 37 °C in YPD medium supplemented with osmotic stabilizers.

To investigate whether the increase in cell size would impair yeast growth, we further measured the growth curves of the wild type and *xrn1*∆ strains. The growth of the *xrn1*∆ mutant was slightly decreased at 28 °C compared to the WT. However, it was significantly decreased at 37 °C (Figure 3a). The slow-growth phenomenon was not caused by a decrease in the budding rate of the *xrn1*∆ mutant, because we found that the budding rate of the *xrn1*∆ mutant was not significantly different from that of the wild type (Figure 3b). We also analyzed the growth of the *xrn1*∆ mutant on YPD medium at 37 °C and found that the growth was defective in the *xrn1*∆ strain at 37 °C, which is consistent with previous research [22]. The addition of osmotic stabilizers such as NaCl, KCl and sorbitol did not fully restore the growth defect (Figure 3c). In addition, there was no significant difference in the growth of medium containing KCl, NaCl and sorbitol at 28 °C between the *xrn1*∆ and wild-type (Figure S3).

### 3.3. XRN1 Deletion Altered the DNA Content and Led to Hypersensitivity to Nocodazole

**Figure 4:**
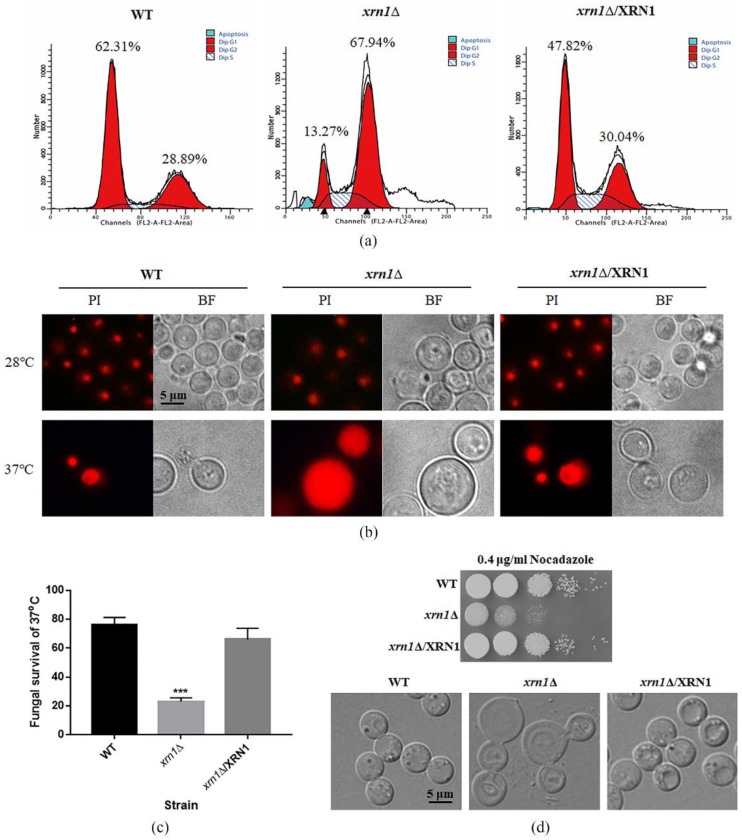
*XRN1* deletion altered DNA content and resulted in hypersensitivity to nocodazole. (**a**) Determination of DNA content by flow cytometry. In total, 20,000 cells were collected from each sample for testing. The first red peak on the left of each picture represents cells in G1 phase with DNA content of N. The proportion of cells with DNA content of N in the WT, *xrn1*∆ and complementation strains were 62.31%, 13.27% and 47.82%, respectively. The second red peak represents cells in G2 phase with DNA content of 2N. The proportion of cells with DNA content of 2N in the WT, *xrn1*∆ and complementation strains were 28.89%, 67.94% and 30.04%, respectively. The blue peaks and oblique lines are cells undergoing apoptosis and cells in S phase, respectively. (**b**) Observation of PI-stained nucleus. Yeast cells were cultured in the YPD medium at 28 °C or 37 °C for 60 h. Cells were collected for RNase digestion and PI staining, and fluorescence was observed under a fluorescence microscope. (**c**) Survival of the WT, *xrn1*∆ and complementation strains after 48 h incubation at 37 °C. *** *p* < 0.001; Unpaired *t*-test. (**d**) The response of the *xrn1*∆ strain to nocodazole stress. The lower panel indicates the morphological observation of the yeast cells treated with high concentration (15 μg/mL) of nocodazole for 5 h.

Cell size increases when cell-cycle progression was blocked either by chemical or genetic perturbations [26]. Therefore, we speculate that cell cycle perturbation was involved in expanded cell growth. As expected, the deletion of *XRN1* led to increased DNA content, as revealed by flow cytometry analysis of PI-stained cells. Deletion of *XRN1* resulted in an increase in the G2/M phase cell population (67.94%) compared to the WT and complementation strains (28.89% and 30.04%, respectively) (Figure 4a). This indicates that the DNA replication stage was not influenced by knockout of *XRN1*, but the stage after DNA replication was affected.

In addition, after staining with PI, the aggregated fluorescent was observed in the WT, *xrn1*∆ and complementation strains cultured at 28 °C. However, the *xrn1*∆ mutant showed massively enlarged PI stained nuclei after incubation at 37 °C for 60 h. More than half of the *xrn1*∆ mutant cells exhibited this phenotype. We speculate that a large amount of DNA flowed into the cytoplasm in the *xrn1*∆ strain, so the entire cell was full of fluorescence. The nucleus of WT and complementation strains were still in a nucleation at 37 °C (Figure 4b). At the same time, we determined yeast survival rate and found that the Xrn1-deficient strain died in large numbers, and only 23% of the mutant cells survived, while the survival rates of the WT and complementation strains were 76% and 66%, respectively (Figure 4c). These phenomena indicate that the enlargement of the cells caused by *XRN1* deletion affected the cell cycle progression, blocking the cell cycle in the G2/M phase and resulting in slow growth or even death.

Since microtubules play a pivotal role in organizing the cellular contents and forming the mitotic spindle that is crucial for cell division [27], we tested the response of the *xrn1*∆ strain to nocodazole. Our results showed that the *xrn1*∆ strain was more sensitive to nocodazole than WT and complementation strains, and cell morphology was deformed when the yeast cells were treated with high concentrations (15 μg/mL) of nocodazole for 5 h (Figure 4d). This result indicates that the deletion of *XRN1* might also lead to the abnormal expression of microtubules synthesis-related genes.

### 3.4. Multiple Functions of XRN1 in C. Neoformans

**Figure 5:**
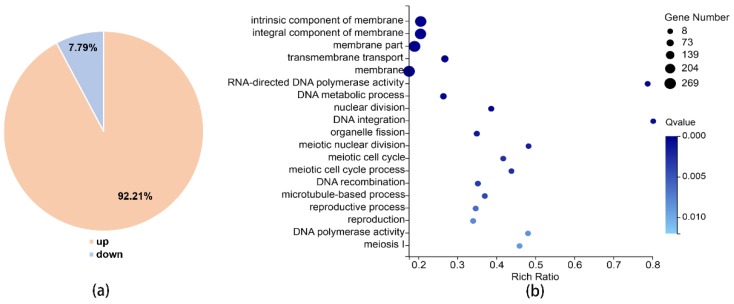
RNA-sequencing results. (**a**) Significantly different genes. (**b**) GO analysis (*p*-value < 0.01).

To further investigate the function of Xrn1 in *C. neoformans*, we used RNA sequencing to detect changes in RNA levels with BGISEQ-500 platform. The average sequencing data reached 21.54 million per sample, and 1.08 Giga clean bases (Gb) in total; the average proportion of filtered reads was 98.16% (Table S2). The cor function in R Project was used to calculate the Pearson’s correlation between two samples. Pearson’s correlation can reflect the similarity of overall gene expression between samples. The higher the Pearson’s correlation, the more similar the gene expression level. Pearson’s correlation between 3 parallel samples from the WT and *xrn1*Δ mutant was greater than 0.98 (Figure S4). This indicates similar gene expression levels between parallel samples. Comparing gene expression levels in the wild-type and *xrn1*Δ mutant, we found that 1271 genes were significantly altered, of which 92.21% were significantly up-regulated and only 7.79% were significantly down-regulated (Figure 5a, Table S3). For Gene Ontology (GO) analysis, *XRN1* was found to be associated with components of membrane, DNA metabolism, integration and recombination, DNA polymerase activity, meiotic cell cycle, nuclear division, organelle fission, microtubule-based process and reproduction etc., (*p*-value < 0.01) (Figure 5b). According to Kyoto Encyclopedia of Genes and Genomes (KEGG) pathway analysis, we found that the differences were significantly enriched in the following pathways: meiosis, MAPK signaling pathway, tryptophan metabolism, amino sugar and nucleotide sugar metabolism, cell cycle, phenylalanine metabolism, hippo signaling pathway, and mitophagy (*p*-value < 0.05) (Table 1). These results showed that the impact of Xrn1 is extensive because it affected the expression of many genes.

### 3.5. XRN1 Deletion Up-Regulated the Expression of Periodic Genes

**Figure 6:**
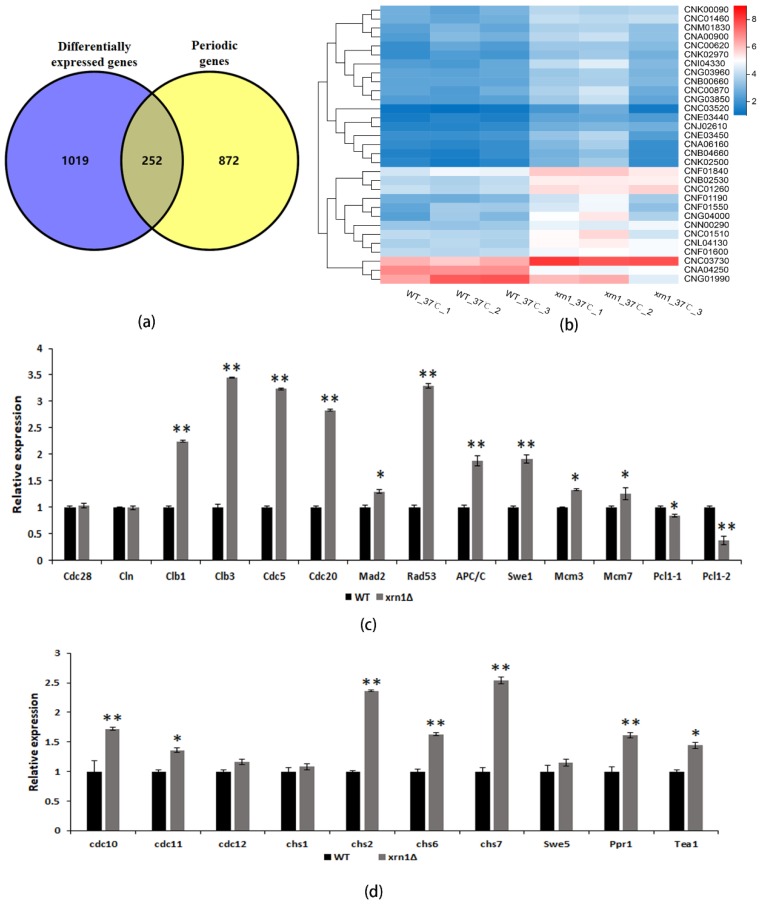
The expression of periodic genes was up-regulated in the *xrn1*∆ strain. (**a**) Overlap between significantly different genes and periodic genes. (**b**) Heatmap of significantly different genes involved in the cell cycle pathway. (**c**) Expression of cell-cycle genes in the WT and *xrn1*Δ mutant. Asterisks indicate significantly different (** *p* < 0.01; * *p* < 0.05; *t*-test). (**d**) Expression of chitin synthase genes and transcription factors in WT and *xrn1*Δ mutant. Asterisks indicate significantly different (** *p* < 0.01; * *p* < 0.05; *t*-test).

In previous studies, 1134 periodic genes were found in *C. neoformans* by investigating transcriptional dynamics of synchronized cells [28]. Since these results came from H99 strain, we used BLASTn software to convert these periodic genes into genes corresponding to JEC21, the comparison parameter E-value was set to 1E-10. By comparing significantly different genes in the *xrn1*Δ strain to periodic genes, we found that the expression of 252 periodic genes (19.8% of the significantly different genes) was significantly different in Xrn1-deficient strains (Figure 6a, Table S4).

DNA replication, spindle assembly, and mitosis genes are highly conserved in a temporal order during fungal cell cycles [28]. We found an increase in the proportion of the *xrn1*∆ strains in the G2/M phase detected by Fluorescence Activating Cell Sorter analysis, so we focused our investigations on spindle assembly and mitosis genes. The cell cycle pathway was altered in transcriptome analysis, and the expression of 29 genes in this pathway was significantly up-regulated, and only 2 genes were down-regulated (Figure 6b). We selected 24 genes for qRT-PCR validation and confirmed that the RNA sequencing results were believable. The expression of *CLB1*, *CLB3*, *CDC5*, *CDC20*, *MAD2*, *RAD53*, *APC/C*, *SWE1*, *MCM3* and *MCM7* genes were up-regulated, and *PCL1* was down-regulated, however, the mRNA levels of *CDC28* and *CLN1* were the same as the WT (Figure 6c). Cytokinesis is the final process of producing identical daughter cells, which required septin, myosin, chitin synthase and other components [29]. We also verified the expression levels of these genes and found that *CDC10*, *CDC11*, *CHS2*, *CHS6* and *CHS7* were all up-regulated, while *CDC12* and *CHS1* were not significantly changed (Figure 6d). Two periodic transcriptional factors were up-regulated in the *xrn1*∆ strain (Figure 6d), including *PPR1* and *TEA1*, which are partially conserved transcription factor in *C. neoformans* [28]. There was no significant difference in the expression of transcription factor *SWE5*.

## 4. Discussion

Xrn1, a conserved 5′-3′ exoribonuclease, is a component of cytoplasmic processing (P) bodies and stress granules that preferentially degrades 5′ monophosphorylated single-stranded RNA [8]. Xrn1 is a key protein for monitoring mRNA level, and its defect often leads to many biological dysfunctions [7]. In our study, the deletion of *XRN1* in *C. neoformans* resulted in a significant increase in cell size and obviously reduced growth rate in liquid YPD at 37 °C (Figure 2 and Figure 3a). However, this slow growth phenomenon was not caused by a decrease in budding rate of exponentially growing cells (Figure 3b). In previous research, *XRN1* has been shown to act as an accessory protein in microtubule function in *S. cerevisiae* [11]. Nuclear migration during budding and separation of the spindle pole body were all mediated by microtubules [11]. Consistent with this result, the *XRN1* mutant of *C. neoformans* showed hypersensitivity to nocodazole, and the cell morphology was deformed under the microscope when the yeast cells were treated with high concentrations of nocodazole for 5h (Figure 4d). These indicate that the microtubule function in Xrn1-deficient strains was affected to some extent.

Cells could adjust their growth and size according to external inputs to comply with specific fates and developmental programs [2]. For larger unicellular organisms, surface transport may be a limiting factor for cell growth. Consistent with previous studies, the *xrn1*∆ strain exhibited a growth defect at 37 °C on the YPD plate, which was partially remediated by providing 0.5 M NaCl, 0.5 M KCl, or 1 M sorbitol as an osmotic support (Figure 3c). This indicates that the growth defect was not entirely due to the imbalance of cation homeostasis or damage to the cell wall. We measured the DNA content in the late log phase by flow cytometry and found that 67.94% cells in the *xrn1*∆ strain were arrested in the G2/M phase (Figure 4a). Consistent with the growth defect on the YPD plates at 37 °C, a large amount of DNA flowed into the cytoplasm when the *xrn1*∆ strain was incubated in the liquid YPD medium at 37 °C for 60 h (Figure 4b). This indicates that the nucleus might have disappeared and was not functioning properly. Uncontrolled exchange between the nuclear interior and cytoplasm leads to DNA damage [30]. In previous reports, Xrn1 affects growth ability at 37 °C, capsule synthesis, laccase activity and mating [22]. In addition, the *xrn1*Δ mutant exhibits reduced virulence in invertebrate models [22]. Our results further confirmed growth defects of *xrn1*Δ mutant at 37 °C and found that the cell became larger and the cell growth rate slowed. After long-term induction at 37 °C, the cell cycle process was blocked in the G2/M phase, DNA diffused into the cytoplasm, and cell deaths occurred.

To explore the role of Xrn1 *in C. neoformans*, we performed RNA sequencing and found that cell cycle pathway was significantly enriched by KEGG analysis, and 19.8% of the significantly different genes were periodic (Figure 6a). Some periodically expressed genes, such as cell cycle-related genes, chitin synthase genes and transcription factors, were significantly up-regulated in *xrn1*∆ strain, which were further verified by qRT-PCR (Figure 6c,d). The highly conserved exonuclease Xrn1 has been proved to regulate gene expression in eukaryotes by coupling nuclear DNA transcription to cytosolic mRNA decay [31]. The absence of Xrn1 up-regulates the expression of other major exonuclease-encoding genes (i.e., Xrn2 and Rrp44), thereby compensating to some extent [32]. However, from the transcriptome data, the expression of these two genes was up-regulated no more than 1.5-fold at 37 °C. In unicellular organisms, it appears that Xrn1 targets specific mRNAs involved in growth and meiosis and the degradation of these target mRNAs cannot be compensated by the exosome [7]. This indicates that the affected periodic genes seem not be compensated by other ribonucleases. However, it was unclear whether these phenotypes were due to the direct or indirect effects of Xrn1 on specific targets based on our existing results.

In *C. neoformans*, the MAPK cascade is involved in a coordinated response to high temperature, which is mediated by the cell integrity pathway and plays an important role in its proliferation process [33]. Deletion of *XRN1* affected the synthesis of chitosan, chitobiose, GlcNAc and chitin in the UDP sugar metabolism pathway. UDP-GlcNAc acts as a substrate in the cell cycle and is involved in the synthesis of 4%–5% chitin in the cell wall. Its increased production can satisfy the higher requirements for amino sugars in M-phase cells [34]. Disruption of the tryptophan metabolism pathway renders the strain unable to survive, and inhibition of this pathway leads to arrest of cryptococcal cell growth [35]. In *Aspergillus fumigatus*, the biosynthesis of aromatic amino acids is an important metabolic pathway, including tryptophan, phenylalanine, and tyrosine [36]. Not only are they necessary for growth, but they are also precursors of several toxins. The “Hippo” signaling pathway, called “Ace2 and morphogenesis (RAM) network regulation”, is related to the separation of mother and daughter cells. Mutants in this pathway exhibit cell separation defects and accumulate large cells [37]. Degradation of dysfunctional mitochondria through the autophagy pathway is important for eliminating ROS and cell survival [38]. The effect of Xrn1 deficiency on MAPK signaling pathway, amino sugar metabolism, amino acid metabolism, Hippo signaling pathway and mitophagy may also be responsible for cell growth defects.

mRNA turnover in yeast is very rapid, with a median half-life of around 2 min [39]. The half-lives of specific mRNAs with short half-lives are increased in the Xrn1-deficient strain [10]. Xrn1 is required at specific points in development to target a specific set of RNAs involved in cell shape change and cell proliferation [7]. However, the essential cell-cycle processes are conserved in periodicity and in timing of the expression [28]. We speculate that deletion of *XRN1* might lead to disorder of cell cycle progression because the periodic genes were not to be degraded in time. Accumulation of mRNAs of these periodically expressed genes will lead to many downstream consequences, including a delayed cell cycle and growth defects at high temperatures.

## 5. Conclusions

The absence of *XRN1* caused an increase in cell size, defective in growth, and altered DNA content at 37 °C. The results of RNA sequencing showed a significant difference in the cell cycle pathway, and the expression of periodic genes such as cell cycle-related genes, chitin synthase genes and transcription factors were up-regulated in the *xrn1*∆ strain. In addition, 19.8% of the significantly different genes were periodic genes, which are conserved in periodicity and in timing of the expression. This gives us a new perspective to explain the effect of Xrn1 on growth and reproduction. Moreover, cell size exceeding the upper limit can cause pathologies, aging and even death. The study of physiological changes in over-sized cells can provide clues to the mechanism of cell senescence.

## Figures and Tables

**Table 1:** KEGG pathway analysis.

Pathway ID ^1^	Pathway Name	Level	Q Value
ko04113	Meiosis-yeast	Cell growth and death	0.000301
ko04011	MAPK signaling pathway-yeast	Signal transduction	0.000342
ko00380	Tryptophan metabolism	Amino acid metabolism	0.003806
ko00520	Amino sugar and nucleotide sugar metabolism	Carbohydrate metabolism	0.006373
ko04111	Cell cycle-yeast	Cell growth and death	0.020641
ko00360	Phenylalanine metabolism	Amino acid metabolism	0.042172
ko04392	Hippo signaling pathway-multiple species	Signal transduction	0.042172
ko04139	Mitophagy-yeast	Transport and catabolism	0.045003

^1^ Pathway with the *p*-value < 0.05 is listed.

## Supplementary Materials

The following are available online at https://www.mdpi.com/2073-4425/11/4/430/s1, Figure S1: Deletion of *XRN1* in *C. neoformans*, Figure S2: Cell morphology at 37 °C, Figure S3: Growth in medium supplemented with osmotic stabilizers, Figure S4: Heatmap of Pearson’s correlation, Table S1: Primers used in this study, Table S2: RNA-sequencing data of the WT and *xrn1*Δ strains, Table S3: Significantly different genes, Table S4: Overlap between significantly different genes and periodic genes.
