# Supplementary material for: Effects of 5′-3′ Exonuclease Xrn1 on Cell Size, Proliferation and Division, and mRNA Levels of Periodic Genes in Cryptococcus neoformans
Source: Genes (Basel). 2020 Apr 16;11(4):430. doi: 10.3390/genes11040430 (PMC7230856; doi:10.3390/genes11040430)
Supplement: Supplementary file 1 [file genes-11-00430-s001.zip › Xrn1-supplementary files-Genes-V1/Xrn1-supplementary materials-Genes-V.docx]

**Supplementary materials**


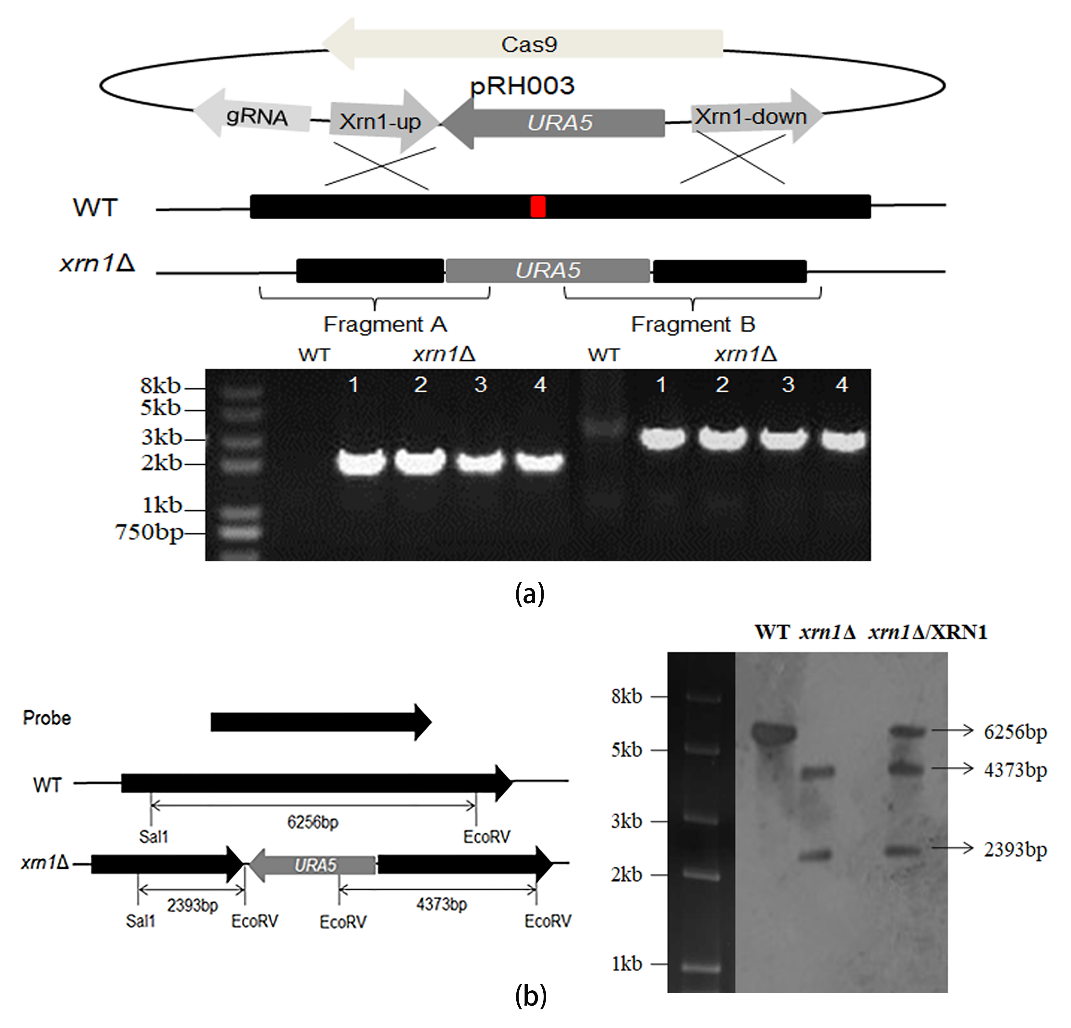


**Figure S1.** Deletion of *XRN1* in *C. neoformans*. **(a)** Xrn1-deficient strain was constructed by replacing with the genomic-copy *URA5* cassette via CRISPR-Cas9 system. Using the CRISPR-Cas9 tool to cause DNA double-strand breaks in the red position and double crossover is mediated by homology-directed repair. Two fragments, 2029 bp and 2845 bp, were obtained in the *xrn1*∆ mutant by PCR, but no PCR product was amplified from the wild-type. **(b)** Southern blotting analysis. The left panel shows the locations of probe, *Eco*RV and *Sal*I in the wild-type and Xrn1-defective genomes. Probe was obtained using primers Xrn1-up-F and Xrn1-down-R. Genomic DNA of the WT, *xrn1*∆ and complementation strains was digested by *Eco*RV and *Sal*I. The right panel shows the results of the Southern blotting. Different bands were detected in the WT, *xrn1*∆ and complementation strains.


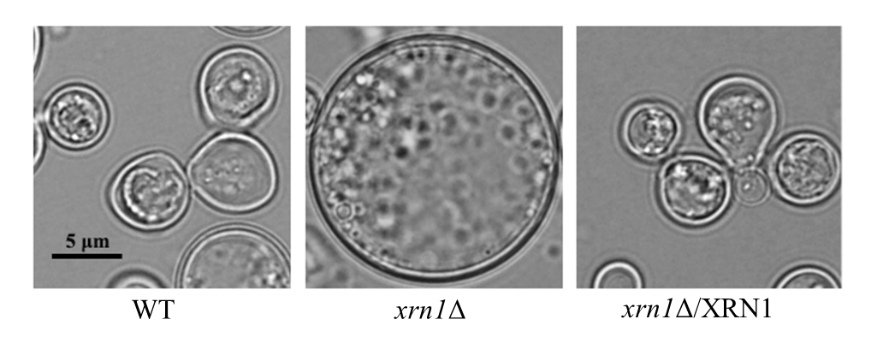


**Figure S2.** Yeast strains were cultured in YPD medium at 37°C for 3 days. Cell morphology was observed under a microscope.


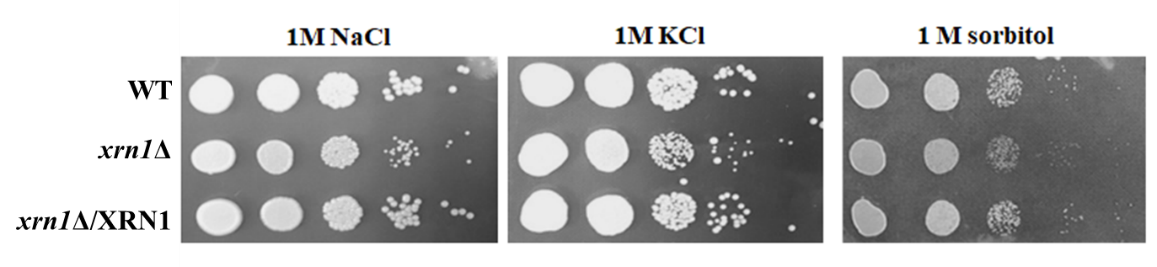


**Figure S3.** Yeast strains were cultured on YPD agar medium containing 1 M NaCl, 1 M KCl, and 1 M sorbitol, and incubated at 28°C for 3 days.


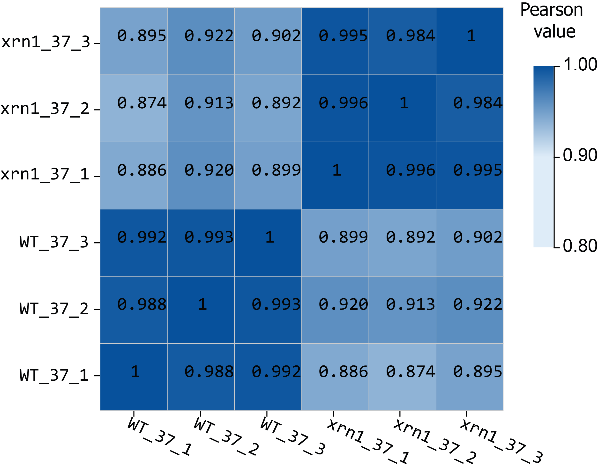


**Figure S4.** Heatmap of Pearson’s correlation. The cor function in R Project was used to calculate the Pearson’s correlation coefficient between every two samples. Pearson’s correlation between 3 parallel samples was greater than 0.98.
